# Low-Energy Electron Irradiation of Tick-Borne Encephalitis Virus Provides a Protective Inactivated Vaccine

**DOI:** 10.3389/fimmu.2022.825702

**Published:** 2022-03-07

**Authors:** Julia Finkensieper, Leila Issmail, Jasmin Fertey, Alexandra Rockstroh, Simone Schopf, Bastian Standfest, Martin Thoma, Thomas Grunwald, Sebastian Ulbert

**Affiliations:** ^1^ Department of Vaccines and Infection Models, Fraunhofer Institute for Cell Therapy and Immunology IZI, Leipzig, Germany; ^2^ Fraunhofer-Institute for Organic Electronics, Electron Beam and Plasma Technology FEP, Dresden, Germany; ^3^ Department of Laboratory Automation and Biomanufacturing Engineering, Fraunhofer Institute for Manufacturing Engineering and Automation IPA, Stuttgart, Germany

**Keywords:** vaccine, irradiation, tick-borne encephalitis virus, zoonosis, virus inactivation

## Abstract

Tick-borne encephalitis virus (TBEV) is a zoonotic flavivirus which is endemic in many European and Asian countries. Humans can get infected with TBEV usually *via* ticks, and possible symptoms of the infection range from fever to severe neurological complications such as encephalitis. Vaccines to protect against TBEV-induced disease are widely used and most of them consist of whole viruses, which are inactivated by formaldehyde. Although this production process is well established, it has several drawbacks, including the usage of hazardous chemicals, the long inactivation times required and the potential modification of antigens by formaldehyde. As an alternative to chemical treatment, low-energy electron irradiation (LEEI) is known to efficiently inactivate pathogens by predominantly damaging nucleic acids. In contrast to other methods of ionizing radiation, LEEI does not require substantial shielding constructions and can be used in standard laboratories. Here, we have analyzed the potential of LEEI to generate a TBEV vaccine and immunized mice with three doses of irradiated or chemically inactivated TBEV. LEEI-inactivated TBEV induced binding antibodies of higher titer compared to the formaldehyde-inactivated virus. This was also observed for the avidity of the antibodies measured after the second dose. After viral challenge, the mice immunized with LEEI- or formaldehyde-inactivated TBEV were completely protected from disease and had no detectable virus in the central nervous system. Taken together, the results indicate that LEEI could be an alternative to chemical inactivation for the production of a TBEV vaccine.

## Introduction

Tick-borne encephalitis virus (TBEV) belongs to the family *Flaviviridae* of the genus Flavivirus which includes several major human pathogens such as dengue, Japanese encephalitis, Zika, West Nile, or yellow fever viruses (1). These are enveloped, single positive-stranded RNA viruses which are primarily transmitted by arthropod vectors (2). TBEV is transmitted by ticks and is endemic to several European and Asian countries (3). Its natural hosts are small rodents, but also humans can be infected usually *via* tick bites, although they represent dead-end hosts (4). With several thousand clinical cases per year, it is currently the most important tick-borne virus, and disease symptoms range from fever to neurological complications such as meningitis and encephalitis, which in some cases can be fatal (5). Especially older or immunocompromised individuals are at risk of developing severe forms of illness.

Vaccines for the protection from TBEV induced disease are available and widely used in endemic areas (6, 7). They consist of whole viruses, chemically inactivated by formaldehyde (FA), a technology used for decades to generate vaccines against many different pathogens. Other examples include hepatitis A, seasonal influenza, polio, or Japanese encephalitis viruses (8). Although FA-inactivation is relatively simple and well established, the process bears severe disadvantages: the chemical is toxic, which complicates large-scale production processes and generates problems of waste and residual traces in the final product. The usage of only low amounts of FA leads to the need for long inactivation times of several days or weeks (9). In addition, FA acts by crosslinking and modifying structural components like proteins, hence the treatment has been reported to damage antigenic structures impacting antigenicity of vaccines (8, 10, 11). Other chemicals used to inactivate viruses, mostly the alkylating compound β-propiolactone, have less impact on antigenicity but are also highly hazardous (12). Therefore, there is a need for novel inactivation methods as alternatives to chemical treatment.

A technology for the inactivation of pathogens that does not rely on chemical treatment is ionizing radiation, which primarily destroys nucleic acids, but leaves other structural components largely intact (13). It is known that pathogens inactivated by gamma-, high energy electron or X-radiation can induce highly protective immune responses (14–17). However, a major drawback of the existing radiation technologies is the requirement of complex shielding constructions that limit their use to specialized radiation facilities (18). This is due to the high doses necessary to inactivate viruses, which require and/or generate large amounts of radioactivity. As a consequence, ionizing radiation has not been compatible with pharmaceutical production processes, and viral vaccine candidates developed using irradiation remain experimental until today.

We have previously shown that low-energy electron irradiation (LEEI) overcomes these major limitations of other ionizing irradiation technologies. LEEI consists of electrons accelerated with up to 500 kilo electron volts (keV) and very rapidly delivers high doses necessary for pathogen inactivation, but only requires minimal shielding, which enables its use in standard laboratories (19). Different pathogens have been inactivated using LEEI, and the studies showed the potential of the technology to generate efficient vaccines (20–22). In addition, principles for automated processing of LEEI in a biotechnological production setting have been developed (19). Here, we describe the application of LEEI in such an automated process on TBEV and investigate whether LEEI could be an alternative to chemical inactivation in generating a TBEV vaccine.

## Materials and Methods

### Cell and Virus Culture

BHK-21 cells and Vero E6 cells (DSMZ, Braunschweig, Germany) were propagated in Dulbecco’s modified Eagle’s medium (DMEM, ThermoFisher Scientific, Germany) supplemented with 10% heat inactivated FCS and 1% penicillin/streptomycin (Gibco) at 37°C and 5% CO_2_.

TBEV (Hypr 9BMP U39292.1, kindly provided by Uwe Liebert, Institute for Virology, Leipzig University) was cultivated in BHK-21 cells. Cells were infected with a multiplicity of infection of 0.1 TCID50/cell and incubated for 2 days at 37°C and 5% CO_2_. The virus was purified from cell culture supernatant by ultracentrifugation (30,000 rpm) on a sucrose cushion (15% (w/v) sucrose in PBS) for 3 h at 4°C.

Titration of TBEV was performed by tissue culture infectious dose 50 (TCID50) assay on BHK-21 cells. In short, for the TCID50 assay 10-fold dilutions of viral stocks were incubated on confluent BHK-21 cell monolayers in a 96-well microwell plate for 4 days. The cells were monitored for cytopathic effects (CPE) and the titer was calculated using the Reed-Muench method (23).

### LEEI-Based Inactivation

Virus samples were irradiated as previously described (19). Briefly, irradiation was performed in a custom-built irradiation device situated at the Fraunhofer Institute for Cell Therapy and Immunology (Leipzig, Germany). The device can accommodate different modules that enable an automated LEEI of liquids. A module using disposable bags was used for TBEV irradiation. The bags made of polyethylenterephthalat were filled with 10 ml purified TBEV diluted in PBS containing the stabilizer trehalose at 12% (w/v) and sealed. The filled bags are automatically passed through the irradiation source at a fluid velocity of 50 mm/s. For irradiation the acceleration energy was set to 300 keV and the applied irradiation dose was adjusted by regulating the beam current (in mA). As a control one bag was processed in the module without LEEI (0 kGy). All experiments were performed at 4°C. Afterwards the bags were reopened and the samples were recovered for further experiments.

### Chemical Inactivation

Chemical inactivation of TBEV was conducted according to WHO’s Good Manufacturing Practice guidelines (24). Briefly, purified TBEV in PBS containing 12% trehalose was mixed with formaldehyde to a final concentration of 0.05% (ThermoFisher Scientific, Germany) and incubated at 22°C for 5 days. To remove residual formaldehyde the samples were dialyzed against cold PBS for 2 h. Inactivation was verified as described in section *Verification of TBEV Inactivation*.

### Verification of TBEV Inactivation

To identify the LEEI-dose necessary for complete inactivation of TBEV, irradiated samples were analyzed on BHK-21 cells seeded in 6-well cell culture plates one day prior to infection. The cells were inoculated with 100 µl of irradiated or active TBEV as a positive control per well (in duplicates). Mock-infected wells served as a negative control. Cells were observed for the appearance of cytopathic effects (CPE) over 3 days. Cell culture supernatants were then passaged onto fresh cells and observed for another 3 days. The sample was regarded as inactivated when no CPE was visible after passage. Samples with visible CPE were titrated in a TCID50 assay to quantify the titer reduction of infectious virus caused by LEEI.

A second experiment was conducted to re-confirm the inactivation of TBEV using 20 kGy. Active TBEV served as a positive control and mock-infected cells as a negative control. BHK-21 cells seeded in 6-well cell culture plates were inoculated with virus samples (in triplicates). After adsorption at 37°C for 1 h, the virus-containing medium was collected, cell monolayers were washed with PBS to completely remove unadsorbed virus and incubated further in fresh cell culture medium for 3 days. Cell culture supernatants were then passaged twice onto fresh cells and incubated for another 3 days per passage. After each passage, 140 µl of cell culture supernatant was used for the extraction of viral RNA using QIAamp viral RNA mini kit following manufacturer’s instructions. Isolated RNA was then analyzed using RT-qPCR as described in Quantification of Viral RNA and Infectious Virus in Organ Homogenates.

### ELISA

The influence of either LEEI-based or formaldehyde inactivation on the antigenicity of TBEV compared to the non-treated controls was tested by an indirect enzyme-linked immunosorbent assay (ELISA). In short, inactivated TBEV samples were coated on a NUNC polysorp 96-microwell plate (ThermoFisher Scientific, Germany) in coating buffer (35 mM Na_2_HCO_3_ /15 mM Na_2_CO_3_, pH 9.6), in a total volume of 100 µl per well overnight at 4°C. The plate was washed three times in PBS containing 0.05% (v/v) Tween20 (PBS-T) and wells were blocked with 5% (w/v) skim milk powder in PBS. Sera from two patients tested positive for TBEV by virus neutralization tests (kindly provided by Luisa Barzon, Padova University, Italy, with approval from the local ethical review board) were diluted 1:100 in 100 µl 5% (w/v) skim milk in PBS and added for 2 h at room temperature. After another wash step the plate was incubated with a secondary HRP-conjugated goat anti-human IgG antibody (1:20,000, Dianova, Hamburg, Germany) for 1 h at room temperature. The plate was washed again and TMB-ELISA substrate (Biozol, Eching, Germany) was added for 30 min at room temperature. The reaction was stopped by addition of 1M H_2_SO_4_. Absorbance was measured at 450 nm and 520 nm reference wave length in a standard ELISA reader (Infinite M200, Tecan, Männedorf, Switzerland).

For the analysis of TBEV-binding antibodies, active TBEV was coated on NUNC PolySorp 96-microwell plates overnight at 4°C. Heat inactivated (56°C for 30 min) sera from the immunized mice were diluted 1:100 in 5% skim milk in PBS, 50 µl were added to each well in duplicates and binding mouse IgG were detected with a HRP-conjugated rabbit anti mouse IgG antibody (1:2000, Dako, Glostrup, Denmark). To test for the avidity of TBEV-binding antibodies in the mouse sera, an additional 3 min washing step using 7 M urea in PBS-T was included after serum incubation. The denaturing urea treatment disrupts weak antibody-antigen complexes and by that dissociates antibodies binding with low affinity. An identical plate without the urea wash served as a reference to calculate the relative avidity with the following formula:


$$relative\;avidity[\%]=\frac{OD_{450/520\;nm}with\;7M\;urea\;wash}{OD_{450/520\;nm}without\;urea\;wash}\times 100$$

### Virus Neutralization Assay

TBEV neutralizing antibodies in mouse sera taken 1 week after the second and third immunization were measured in a focus reduction neutralization test (FRNT). The sera were serially diluted and incubated with 80 focus forming units (FFU) of purified TBEV for 1 h at 37°C. The virus-serum mixture was transferred to Vero E6 cell monolayers in 96-well microwell plates and incubated for another hour. Cells were overlaid with 1% methylcellulose in DMEM with 2% FCS and 1% penicillin/streptomycin and incubated for 2 days. The cells were fixed with 4% formaldehyde in PBS and permeabilized in Perm-Wash buffer (0.1% Saponin and 0.1% BSA in PBS). Immunostaining of TBEV foci was performed using the primary flavivirus antibody 4G2 (Absolute antibody, Oxford, UK) as described previously (25). Neutralizing antibody titer was defined as the reciprocal of the last serum dilution that showed a minimal 50% reduction of TBEV foci compared to sera from sham-immunized mice. Each experiment was performed in duplicates.

### TBEV Immunization and Challenge Experiment

All animal experiments were carried out in accordance with the EU Directive 2010/63/EU for animal experiments and were approved by local authorities (No.: TVV 01/20). Female BALB/c mice (8 weeks old) were obtained from Charles River (Germany) and kept in a specific pathogen-free environment in isolated ventilated cages. Groups of eight mice were immunized three times at a two-week interval by intramuscular (i.m.) injection into the hind limbs of 100 µl (2 sites, 50 µl each) of 1:1 mixture of Alhydrogel (aluminum hydroxide gel adjuvant, 10 mg/ml aluminum, InvivoGen, France) with either LEEI- or formalin-inactivated TBEV at a dose of 10^6^ TCID50. The immunization schedule with three doses was based on the study by Salat et al. (26), which used the same viral and animal strains. Control mice were sham immunized with vehicle solution (1:1 mixture of Alhydrogel and PBS + 12% trehalose). Blood was collected from the retrobulbar venous sinus at three time points: one week prior to the first immunization and one week after the second and the third immunization. After collection, blood samples were incubated at room temperature for 30 min and centrifuged at 8000 g for 10 min to obtain serum for analysis of TBEV-binding and neutralizing antibodies. Two weeks after the third immunization, all mice were challenged with 100 µl containing 4.4 x 10^4^ TCID50 of purified TBEV *via* intraperitoneal injection. Clinical development of disease was monitored daily for 14 days post-infection and score points were given according to the following criteria: body weight loss (0 points= no weight loss, 5 points= 8-10%, 10 points= 11-19%, 20 points= weight loss ≥20% of initial weight); fur condition (0 points = shiny and clean coat, 2 points = piloerection, 5 points = ruffled fur); eye appearance (0 points= open healthy eyes, 5 points= mildly inflamed, 10 points = highly inflamed and closed); gastrointestinal symptoms due to distention of the intestine (0 points= no symptoms, 5 points= mild, 10 points= moderate abdominal swelling); body posture (0 points= normal posture, 20 points= hunched body posture); activity level and motor function (5 points= slightly reduced activity and reaction, 10 points= coordination disorder and reduced activity, 20 points= apathy and morbidity); neurological symptoms (5 points= mild paralysis of one limb, 10 points= mild paralysis of two limbs, 20 points= complete paralysis of two limbs). Humane endpoints requiring euthanasia were defined as reaching a cumulative score points of 20 for a period of 24 h. Animals acquiring cumulative score points greater than 20 were immediately euthanized.

### Quantification of Viral RNA and Infectious Virus in Organ Homogenates

Brains and spinal cords were isolated and homogenized in gentleMACS™ M Tubes (Miltenyi Biotec, Germany) containing 2 ml of ice-cold PBS using gentleMACS Dissociator (Miltenyi Biotec, Germany). Homogenized tissues were cleared of debris by centrifugation at 2000 g and 4°C for 5 min. RNA was isolated from 140 µl of homogenate supernatants using QIAamp-Viral-RNA-Mini Kit (Qiagen, Germany) according to the manufacturer’s instructions. 5 µl of isolated RNA was reverse transcribed and analyzed with the QuantiTect probe RT-PCR kit (Qiagen, Germany) using TBEV forward primer (5’-GGGCGGTTCTTGTTCTCC-3’), TBEV reverse primer (5’-ACACATCACCTCCTTGTCAGACT-3’) and TBEV probe (5’-TGAGCCACCATCACCCAGACACA-3’) labeled with 6-FAM at the 5’ end (27). 10-fold dilutions of plasmid DNA containing the TBEV-target sequence were used as standards for the quantification of viral genome copy numbers in mouse samples. The presence of PCR inhibitors in mouse brain and spinal cord homogenates was excluded by performing an RNA isolation from homogenate samples with the addition of an internal RNA extraction control provided in genesig^®^ TBEV advanced kit (Primerdesign™ Ltd, UK). The quantity of added internal RNA control detected by qPCR was within the normal range (Cp values of 28 ± 3) according to the information provided by the manufacturer.

For the detection of infectious virus in brain and spinal cord homogenates, Vero E6 cells were seeded at 2.5 x10^4^ cells/well in a 96-well plate in 200 µl of DMEM, 10% FCS, 1% penicillin/streptomycin one day before infection. Next day, the medium was changed to DMEM, 1% penicillin/streptomycin. Serially diluted homogenates were applied in duplicates onto the cells and exchanged for medium after one hour. After two days, TBEV- foci were visualized using immunostaining as described in *Virus Neutralization Assay* section and infectious viral titers were calculated in FFU/ml of homogenate.

### Statistical Analysis

Statistical analysis was performed with GraphPad Prism 6.0.7 (GraphPad Software, Inc., La Jolla, CA, USA). Data were checked for normality using the Shapiro-Wilk test. Normally distributed data of the antibody avidity was analyzed using an unpaired t-test. For analyzing not normally distributed antibody data, a Mann-Whitney-U-test was applied. Differences between groups in clinical score and weight loss on each day post-infection and differences in viral loads were analyzed by a Kruskal-Wallis-test followed by a Dunn’s *post-hoc* multiple comparison test. Level of significance is indicated with * = p < 0.05, ** = p < 0.01, *** = p < 0.001.

## Results

### LEEI-Based Inactivation of TBEV

TBEV was purified from cell culture supernatant and treated in liquid solution with different doses of LEEI to determine the dose necessary for complete inactivation of the virus. Irradiation with 10 kGy reduced the amount of TBEV to a titer below the detection limit, but a CPE was still visible after one passage in the inactivation test. A complete viral inactivation resulting in no detectable infectious virus in cell culture after passage was achieved by application of 20 kGy (**Table 1**).

**Table 1:** Testing of TBEV inactivation with different doses of LEEI; the detection limit is 100 TCID50/ml.

LEEI dose [kGy]	CPE after 1 passage	Titer of active TBEV [TCID50/ml]
0	+	5 x 10^6^
10	+	< 10^2^
20	–	< 10^2^
30	–	< 10^2^

**Figure 1:**
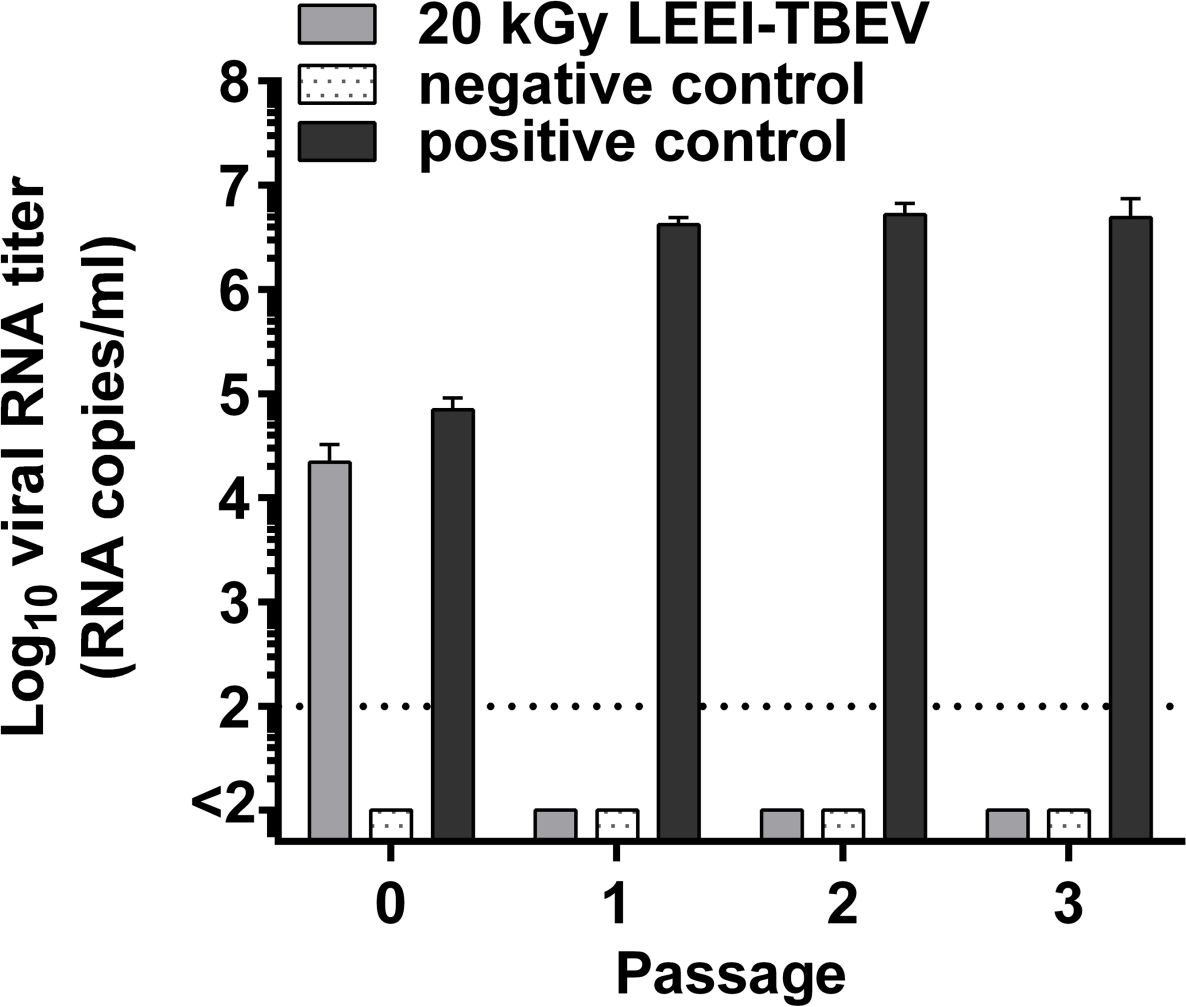
Confirmation of inactivation of LEEI-TBEV samples used for mice immunization. Virus inactivation was performed using a LEEI dose of 20 kGy. Samples were transferred in triplicates to BHK-21 cells and after 3 days the supernatant was passaged onto fresh cells, followed by another passage three days later. Negative control= mock-infected cells; positive control= active TBEV undergoing the LEEI process without irradiation; P0 samples collected at 1 hour post-inoculation; P1= supernatant collected 3 days after inoculation; P2= supernatant collected 3 days after the first passage; P3= supernatant collected 3 days after the second passage. Collected cell culture supernatants were analyzed for TBEV RNA by RT-qPCR. Shown are mean values of triplicates ± standard deviations. The dotted line indicates the limit of detection (100 viral RNA copies).

The inactivating dose of 20 kGy was re-confirmed by two rounds of passages of the irradiated material on cells and the measurement of viral RNA every three days. TBEV RNA could be detected in the non-treated sample throughout the experiment. However, in the LEEI treated TBEV, viral RNA was only present in the inoculum, but not after passaging the material (**Figure 1**).

### Conservation of Antigenicity Upon Inactivation

**Figure 2:**
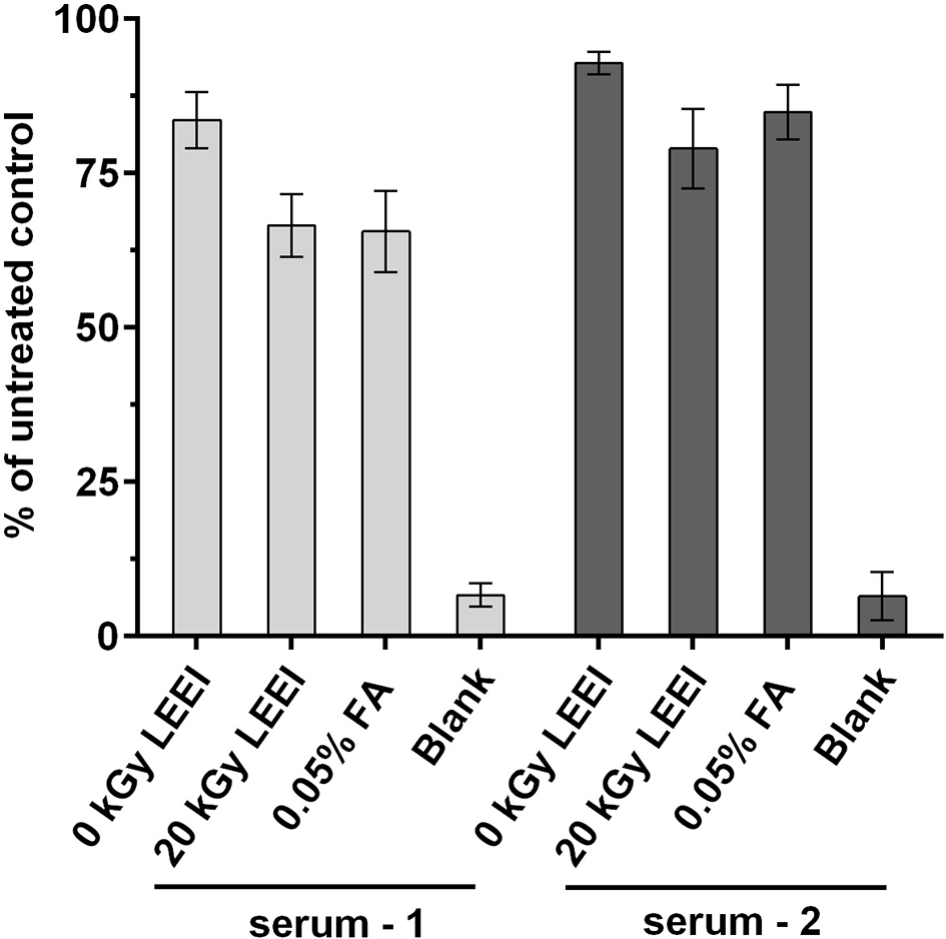
Antigenicity of inactivated TBEV samples. Virus inactivation process was performed either with 20 kGy LEEI or with 0.05% formaldehyde (FA). The ELISA was performed with serum from two TBEV immune individuals. Untreated: virus before inactivation; 0 kGy: virus undergoing the LEEI-process without irradiation; blank: dilution buffer. Shown are mean values of three measurements in duplicates ± standard deviations. Statistical analysis was performed using Kruskal-Wallis test and Dunn’s test of multiple comparisons. No statistically significant differences were detected among the groups.

The impact of LEEI on antigenic structures of TBEV was analyzed in an ELISA assay. Irradiated TBEV samples were investigated and compared to FA-inactivated TBEV, derived from the same viral stock solution. Untreated TBEV virus stock and the non-irradiated sample passing the process (0 kGy) served as controls. Human sera from two individuals infected with TBEV were added to investigate TBEV antigens (**Figure 2**).

A minor loss in antigenicity was caused by the handling of the virus in the automated LEEI-module, independent from irradiation, as evident from the comparison of untreated (set to 100%) and 0 kGy samples (88.2 ± 5.9%). After application of LEEI, a reduction of signal was detected. Antigen integrity after 20 kGy (72.8 ± 8.5%), which leads to complete inactivation of the virus, was on a similar level as the FA inactivated TBEV (75.2 ± 11.7%).

### LEEI and Formaldehyde Inactivated TBEV Elicit Binding and Neutralizing Humoral Immune Responses in Mice

**Figure 3:**
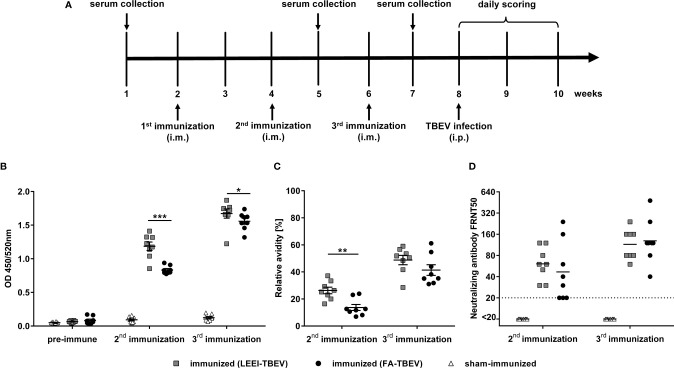
Humoral immune responses after immunization with different inactivated TBEV vaccines. BALB/c mice (n=8) were immunized three times with TBEV inactivated either with 20 kGy LEEI (squares) or 0.05% FA (dots). Sham-immunized mice (triangles) received only buffer and adjuvant. **(A)** scheme of the immunization experiment. **(B, C)** Binding antibodies **(B)** and antibody avidity **(C)** were analyzed in IgG-ELISAs with untreated purified TBEV virions as coating antigen. For avidity measurement, the IgG-ELISA was performed with and without an additional urea wash step after the antibody binding. Both signals were compared in order to calculate the relative antibody avidity. Each data point represents an individual mouse of the same experiment. Data derive from two independent ELISA-assays, each serum sample measured in duplicates per run. Mean values of the groups ± standard error of the mean (SEM) are indicated. **(D)** Neutralizing activities of mouse sera were measured in focus reduction neutralization tests. The dotted line represents the lower detection limit (FRNT50 = 20). Shown are the neutralizing titers of individual mice and the geometric mean of each group. Data derive from two independent FRNT50 assays. All data were tested for normal distribution by a Shapiro-Wilk test. Statistical analysis of normally distributed avidity data was performed by an unpaired t-test. Binding and neutralizing antibodies were analyzed using an unpaired Mann-Whitney U-test (∗p < 0.05; ∗∗p < 0.01; ∗∗∗p < 0.001).

To analyze the potential of LEEI as an alternative inactivation method for providing a safe and immunogenic TBEV vaccine, an immunization study was conducted. TBEV was inactivated either with LEEI or FA and mixed with an aluminium-based adjuvant. Identical titers from the same stock solution were used for both inactivation procedures, in order to ensure that both animal groups received the same amount of inactivated viral particles, equivalent to 10^6^ TCID50. BALB/c mice (eight per group) were immunized three times *via* intramuscular injection according to the immunization scheme shown in **Figure 3A**. Mice immunized with buffer and adjuvant only served as sham-immunized controls. TBEV-specific antibodies could be detected in the blood of all immunized mice one week after the second vaccination (**Figure 3**). The value of absorbance corresponds to the amount of TBEV-specific IgG in the sera which bind to antigens on the whole virus. The antibody titers in mice receiving LEEI-inactivated TBEV were significantly higher (unpaired Mann-Whitney U-test; P=0.0006) than those of animals immunized with FA-inactivated TBEV (**Figure 3B**). The third immunization further increased the level of TBEV-binding antibodies in all immunized animals and the difference remained statistically significant (unpaired Mann-Whitney U-test; P=0.028). At any time points no TBEV-specific antibodies could be detected in the control animals.

Additionally, the avidity of the TBEV-binding antibodies was analyzed by measuring the release of TBEV-bound antibodies caused by the treatment with urea as a chaotropic agent in an ELISA assay (**Figure 3C**). The data show a similar pattern compared to the TBEV-binding antibodies. Antibody avidity in the sera of the LEEI-group was significantly higher (unpaired t-test; P=0.0019) after the second immunization than in the group immunized with FA-treated TBEV. The third immunization led to an increase in the antibody-avidity in all mice of both vaccinated groups. The superiority of the antibody-avidity in the LEEI- over the FA-group was still visible after the third immunization, however, without statistical significance (unpaired t-test; P=0.178).

Next, induction of neutralizing antibodies after the second and third immunizations was evaluated using a focus-reduction neutralization test (FRNT) (**Figure 3D**). After the second immunization, sera from all LEEI-TBEV immunized mice showed neutralizing activity with an average neutralizing titer of 70, whereas three of the mice immunized with FA-inactivated TBEV had neutralizing titers near the detection limit (FRNT50 = 20). The third immunization increased the neutralizing titers in both immunized groups to a similar level of mean titers of 120. Serum of sham-immunized mice showed no detectable neutralizing activity at both investigated time-points.

### LEEI- and Formaldehyde Inactivated TBEV Vaccine Protect Mice Against TBEV Challenge

**Figure 4:**
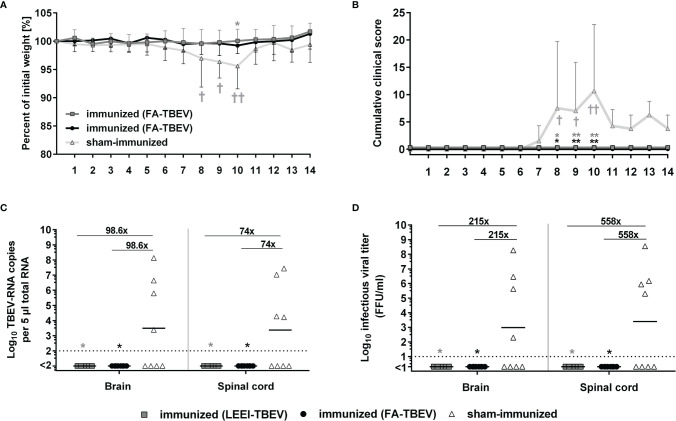
Protective efficacy of inactivated TBEV vaccines against viral-challenge in mice. Immunized mice were infected two weeks after the third immunization with active TBEV. Mice were monitored daily for 14 days post-infection for body weight **(A)**, and clinical score **(B)**. Mice were euthanized upon reaching humane endpoints (marked by a cross) or latest at day 14 post-infection. Body weight and cumulative clinical score are presented as means ± standard errors. After euthanasia, brains and spinal cords were collected and homogenized for viral RNA load analysis using RT-qPCR **(C)** and quantification of infectious virus by a focus-forming assay **(D)**. Shown are viral RNA copy numbers and infectious viral titers of individual mice, geometric means of the groups, and viral reduction compared to the sham-immunized group. Dotted lines indicate limit of detection (= 100 viral genome copies in C and 10 FFU/ml in D). Statistical evaluation of all data was performed using Kruskal-Wallis test and Dunn’s pairwise multiple comparison test. The analysis of weight loss and clinical score was performed for each day post-infection and the statistically significant differences are indicated at the corresponding days (∗p < 0.05; ∗∗p < 0.01).

In order to assess the protective efficacy of the LEEI-inactivated TBEV vaccine, immunized mice were challenged with active TBEV two weeks after the third immunization. Mice were monitored for clinical score and weight loss for 14 days following virus challenge. Starting from 7 days post-infection, sham-vaccinated mice developed first clinical symptoms, like ruffled fur and reduced activity, and began to lose weight. In the following days, the clinical symptoms started to worsen. The symptoms ranged from increased weight loss (up to 11%), abdominal swelling caused by distention of the intestine, ocular inflammation to impaired movement and lethargy, which led to euthanasia of four mice of the control group according to humane endpoints. The remaining four sham-immunized mice all showed symptoms of illness, but were able to survive the infection, which resulted in a 50% (n=4/8) survival rate in the control group. In contrast, none of the mice vaccinated either with LEEI- or FA-inactivated TBEV showed clinical symptoms or weight loss during the entire study duration of 14 days, and all of them survived the infection (**Figures 4A, B**). The differences in cumulative clinical score of both vaccinated groups in comparison to the control vaccinated were statistically significant at day 8, 9 and 10 post-infection (Kruskal-Wallis and Dunn’s test; P values= 0.0105, 0.0053 and 0.0021, respectively). Upon reaching humane endpoints or the latest on day 14 post-challenge, mice were euthanized, and homogenized brains and spinal cords were used to determine viral load by quantitative RT-PCR and virus growth assays (**Figures 4C, D**). Viral RNA copy numbers remained below the detection limit (100 viral genome copies) in all vaccinated mice, with no differences between both vaccinated groups. In addition, 50% of the sham-immunized control mice that had to be euthanized due to reaching defined humane endpoints showed high viral RNA loads at the time of euthanasia in both brain and spinal cord samples. The surviving mice of the control group had no detectable viral RNA at the end of the experiment. The geometric means of viral RNA load in the brains and spinal cords of all control vaccinated mice were 9.9 x 10^3^ and 7.4 x10^3^ genome copies in the tested material, respectively. Viral RNA reduction in both vaccinated groups compared to the control vaccinated mice was over 90-fold in the brain samples and 70-fold in the spinal cord samples (Kruskal-Wallis and Dunn’s test; P=0.0105). Similar significant reduction (Kruskal-Wallis and Dunn’s test; P=0.0105) in infectious viral titers were observed in organ homogenates of vaccinated mice (all below the limit of detection of 10 FFU/ml) in comparison to the control group that showed geometric means of infectious virus of 2.1 x 10^3^ and 5.6 x10^3^ FFU/ml in brains and spinal cords, respectively.

## Discussion

In this study, we analyzed the potential of LEEI as an alternative to chemical treatment for the production of an inactivated TBEV-vaccine. In contrast to other technologies for ionizing radiation, LEEI does not need heavy shielding constructions and therefore has the potential for integration into pharmaceutical production facilities. The major challenge of LEEI is the low penetration depth of the low-energy electrons, which has limited its use so far mainly to the sterilization of surfaces (28). By developing processes to transform liquids into thin films for an automated application, this problem was solved and LEEI was made applicable for the inactivation of pathogens in solution (19). TBEV was treated with LEEI of different doses, and 20 kGy were found to inactivate the virus completely. This is in line with the related flavivirus Zika virus, for which an LEEI-dose of 20 kGy for a complete inactivation was also established (19). 20 kGy was also reported in a previous publication on gamma-irradiated TBEV (29). However, another study found 50 kGy of gamma-radiation to be required for complete inactivation (30). The discrepancy is likely to be based on different irradiation conditions, such as media or temperature. It has been shown that sensitivity to ionizing radiation increases at higher temperatures (31). Hrusková and this study used samples at room temperature or 4°C, respectively. Feldmann et al., irradiated the virus on dry ice.

When compared to the currently used production process of FA-inactivation, LEEI has important advantages. It does not require any toxic chemicals, and the process is much faster. In the setting presented here, 10 minutes were sufficient to inactivate 10 milliliters of TBEV. Using a recently developed continuous irradiation technology, even multi-liter scale preparations could be inactivated within hours (19). This represents a significant improvement over the 5 days inactivation with FA and would substantially speed up the vaccine production process.

In accordance with previously reported data from other LEEI inactivated pathogens, the antigenicity of TBEV was not substantially changed by the radiation. Consequently, when administered to animals, the LEEI-inactivated virus induced detectable TBEV-binding antibodies. The titers were significantly higher in the mice receiving the LEEI-inactivated TBEV than in the group immunized with FA-inactivated virus. After the second immunization also the avidity of the antibodies, which has been shown to correlate with protection (7, 32), was significantly higher after immunization with LEEI-inactivated TBEV. These observations could be explained by the better preservation of antigens in their natural confirmation during the irradiation-based inactivation process. The impact of FA on the antigenicity of vaccine preparations of several pathogens is known, and also for TBEV it has been demonstrated that treatment with FA leads to the loss of several epitopes within the envelope protein, the most important antigen for flavivirus vaccines (33).

The protective efficacy of the vaccine preparations was analyzed in a viral challenge experiment. When infected with live virus, all animals immunized with LEEI- or FA-inactivated TBEV survived without symptoms, whereas the control mice all developed a clinical score, and 50% of them did not survive. In addition, no viral RNA or infectious virus was found in the brains or the spinal cords of vaccinated animals. Mice immunized with either LEEI- or FA-inactivated TBEV were thus completely protected from virus challenge. On the other hand, it was therefore not possible to detect significant differences in vaccine efficacy between the two inactivation methods. It remains to be determined whether the higher avidity of the antibodies induced by LEEI after two immunizations increases protection over chemically inactivated TBEV. A way to address this further would be the usage of a more stringent challenge and/or less antigen per dose. In addition, an immunization schedule of only two doses could be employed.

Taken together, we show that LEEI efficiently inactivates TBEV while maintaining its antigenic properties. Upon vaccination, LEEI-inactivated TBEV induces antibodies of high avidity and protects animals from symptomatic infection and viral load in the CNS. The results indicate that LEEI is worth being further evaluated as an alternative to chemical inactivation, in order to eventually avoid the usage of hazardous substances for the production of inactivated TBEV vaccines.

## Data Availability Statement

The original contributions presented in the study are included in the article/supplementary material. Further inquiries can be directed to the corresponding author.

## Ethics Statement

The animal study was reviewed and approved by Landesdirektion Sachsen, Saxony, Gemany.

## Author Contributions

SU, TG, LI, and JuF designed the study. JuF and LI performed the virus and animal experiments. SS, JuF, BS, and MT performed irradiation experiments. JuF, LI, TG, JaF, AR, and SU analyzed and interpreted the data. SU, JuF and LI wrote the paper. All authors contributed to the article and approved the submitted version.

## Conflict of Interest

SU is co-author on the patent WO 2015011265, which describes the inactivation of viruses by low-energy electron irradiation.

The remaining authors declare that the research was conducted in the absence of any commercial or financial relationships that could be construed as a potential conflict of interest.

## Publisher’s Note

All claims expressed in this article are solely those of the authors and do not necessarily represent those of their affiliated organizations, or those of the publisher, the editors and the reviewers. Any product that may be evaluated in this article, or claim that may be made by its manufacturer, is not guaranteed or endorsed by the publisher.
